# Transaminases are Potential Biomarkers of Disease Severity in COVID-19 Patients: A Single-Center Experience

**DOI:** 10.7759/cureus.11555

**Published:** 2020-11-18

**Authors:** Pravallika Chadalavada, Vinay Padbidri, Rajat Garg, Mohammad Alomari, Arslan Babar, Tariq Kewan, Keerat R Ahuja, Jose Contreras, Mohammed J Al-Jaghbeer, Madhusudhan R Sanaka

**Affiliations:** 1 Internal Medicine, Cleveland Clinic, Cleveland, USA; 2 Internal Medicine, Cleveland Clinic Fairview Hospital, Cleveland, USA; 3 Internal Medicine, Cleveland Clinic Foundation, Cleveland, USA; 4 Critical Care Medicine, Cleveland Clinic Foundation, Cleveland, USA; 5 Pulmonary and Critical Care, Cleveland Clinic, Cleveland, USA; 6 Gastroenterology and Hepatology, Cleveland Clinic Foundation, Cleveland, USA

**Keywords:** sars- cov2, coronavirus disease-2019, gastrointestinal, hepatic manifestations, outcomes

## Abstract

Background: Considering the rapid spread of severe acute respiratory syndrome coronavirus 2 (SARS-CoV2), the clinical implications of gastrointestinal (GI) and hepatic manifestations of coronavirus disease 2019 (COVID-19) in the U.S. population require analysis.

Methods: We retrospectively reviewed all adult patients with COVID-19 admitted to our facility. Patients were divided into two groups based on the presence of GI symptoms and transaminitis at presentation. Univariable analysis was performed to assess the differences between study groups. Kruskal-Wallis and Pearson's chi-square tests were used to compare the median of continuous and categorical variables, respectively. Multivariate logistic regression analysis was performed to identify predictors of mechanical ventilation, cytokine release syndrome (CRS), and mortality after adjusting for baseline variables.

Results: A total of 84 patients were analyzed. After adjusting for baseline comorbidities, presence of GI symptoms (aOR, adjusted odds ratio 4.2, 95% CI, 1.17-15.60, p=0.03) and transaminitis on admission (aOR 5.69, 95% CI, 1.47-21.99, p=0.01) were associated with CRS. Transaminitis on admission and elevated total bilirubin during hospitalization were associated with an increased need for mechanical ventilation (aOR 6.17, 95% CI, 1.49-25.44, p=0.02 and aOR 7.29, 95% CI, 1.73-30.75, p=0.007, respectively). An elevated aspartate aminotransferase (AST) on admission (aOR 13.41, 95% CI, 1.08-165.69, p=0.04) and elevated total bilirubin during hospitalization (aOR 82.68, 95% CI, 1.67-4074.8, p=0.02) were independently associated with an increased risk of mortality in COVID-19 patients.

Conclusion: COVID-19 patients with transaminitis on admission had a higher risk of requiring mechanical ventilation and developing CRS. Patients with elevated AST on admission and elevated total bilirubin had higher mortality. Patients with GI symptoms did not have worse outcomes.

## Introduction

The first outbreak of novel coronavirus (severe acute respiratory syndrome coronavirus 2, SARS-CoV2) occurred in Hubei province, Wuhan, China, in December 2019. Soon after, this virus has rampantly spread across the globe, and the coronavirus disease 2019 (COVID-19) was declared a public health emergency of international concern by the World Health Organization (WHO) on 30th January 2020. As of May 2020, a total of 1,385,834 laboratory-confirmed cases have been documented in the United States. Although the SARS-CoV2 is predominantly a respiratory virus, there is an ongoing investigation on the degree of gastrointestinal (GI) and hepatic involvement of this novel virus.

Emerging studies have highlighted that the angiotensin-converting enzyme 2 (ACE2) receptors not only act as potential binding sites for the SARS-CoV2 but also contribute to enhancing the progression of thrombotic and inflammatory processes [1]. These receptors are also found abundantly in the GI and biliary tract, thus raising suspicion for the GI epithelium and cholangiocytes to be additional sites of viral replication [2]. Mounting evidence from a few studies confirms the GI tropism of SARS-CoV2 by detection of this virus in stool samples of affected patients, thus providing a potential explanation for the GI symptoms, recurrence, and transmission from fecal shedding [3]. These findings were further reaffirmed by another study which reported that the SARS-CoV2 RNA was detected in the stool samples of 83.3% of patients with a mild infection for up to a month [4].

A study from Zhejiang province, China reported that 11.4% of the patients who tested positive for COVID-19 had at least one GI symptom (nausea, vomiting, or diarrhea) at presentation [5]. Strikingly, a significantly higher percentage of patients with GI symptoms developed severe disease when compared to those without GI symptoms. Additionally, 14.8%-78% of the patients with COVID-19 also had abnormal alanine aminotransferase (ALT) and aspartate aminotransferase (AST) accompanied by mildly elevated bilirubin level on admission [6]. Recent studies have suggested that the presence of liver injury on presentation was associated with a significantly higher rate of ICU admission and death [7-8].

Given the rapid spread and high pathogenic capacity of SARS-CoV2 infection, we determined that a more detailed analysis of the clinical characteristics, severity, and implications of GI and hepatic manifestations of COVID-19 is required. Additionally, we also aimed to evaluate the trend of elevation of liver enzymes during hospitalization and compare the overall outcomes among patients with and without GI/hepatic manifestations.

## Materials and methods

Patients

We retrospectively reviewed the electronic medical records of all patients who were diagnosed with COVID-19 and consecutively admitted to Cleveland Clinic Fairview Hospital between March 13th, 2020 and May 1st, 2020. All patients above the age of 18 years with a positive reverse-transcriptase-polymerase-chain-reaction (RT-PCR) assay for SARS-CoV2 at admission were included in this study. This study was approved by the institutional review board of Cleveland Clinic.

Study variables and assessments

We retrospectively collected the following data on all our included patients: age, sex, race and ethnic group, smoking status, comorbidities, clinical symptoms, first recorded vital signs on presentation, first recorded inpatient laboratory tests, oxygen requirement, diagnostic workup, treatment received, complications, and outcomes.

Evaluation of gastrointestinal symptoms

All patients with at least one of the following symptoms, such as anorexia, nausea, vomiting, diarrhea, or abdominal pain at presentation, were considered positive for GI symptoms. We recorded each of these symptoms on the first day of presentation to offset the effect of antibiotics or antivirals, which are usually started during hospitalization. Diarrhea was defined by the presence of more than three loose stools per day. Patients underwent additional testing with stool cultures, stool enteric panel, and stool PCR for *Clostridium difficile* to evaluate for other causes of diarrhea depending on food history, residence, occupational exposure, recent and remote travel, and antibiotic use.

Evaluation of transaminitis

All patients with at least one of the following: elevated ALT or AST > 1 x upper limit of normal (ULN) were considered to have transaminitis. All the included patients had normal baseline liver function tests except one patient with liver cirrhosis who had fluctuating liver enzymes over the past several years. These laboratory parameters were recorded on admission, precluding the influence of other medical therapy and external factors. We also collected the peak AST, ALT, alkaline phosphatase (ALP), total bilirubin, and albumin levels during hospitalization to evaluate the trend of elevation of liver enzymes.

Treatment for COVID-19

On admission, all patients with COVID-19 were started on hydroxychloroquine with a loading dose of 400 mg twice daily, followed by 200 mg twice daily for additional five days and azithromycin 500 mg per day for five days, unless contraindicated. An electrocardiogram was obtained for each patient to check the corrected QT interval at baseline. Tocilizumab was given to COVID-19 patients at the discretion of an infectious disease specialist, in the presence of hypoxia, lung infiltrates on chest radiography, elevated inflammatory biomarkers (either C-reactive protein >3 g/dL or ferritin >400 ng/mL), and concern for clinical deterioration. Tocilizumab was administered as a single dose of 400 mg, and it was given as a 60-minute single IV infusion. Tocilizumab was not given to patients with confirmed or suspected bacterial or fungal infections, platelets count <100,000/mm3, neutrophil count <2000/mm3, and ALT or AST more than five times the ULN range (200 IU/L).

Outcomes

Primary outcomes of interest were ICU admission, development of circulatory shock requiring vasopressor support, development of acute respiratory distress syndrome (ARDS), development of cytokine release syndrome (CRS), development of acute kidney injury (AKI) requiring renal replacement therapy, need for mechanical ventilation, and mortality. Secondary outcomes of interest were the hospital length of stay, deep vein thrombosis (DVT), duration of vasopressor support, and duration of mechanical ventilation.

Statistical analysis

Continuous variables were presented as median (interquartile range) and categorical variables as counts and frequency. Univariable analysis was performed to assess differences between study groups. Kruskal-Wallis test was used to compare medians of continuous variables. Pearson's chi-square tests were used to compare categorical variables. Multivariate logistic regression analysis was performed to identify predictors of mechanical ventilation, CRS, and mortality after adjusting for baseline variables. All statistical analyses were performed using the SPSS software (IBM Corporation, USA). A p-value <0.05 was considered statistically significant.

## Results

Baseline characteristics

Patients With and Without GI Symptoms

A total of 84 patients were included in the analysis. Forty-four (52.3%) patients had GI symptoms. The most common GI symptom was diarrhea (n=29), followed by nausea and vomiting (n=18), loss of appetite (n=12), and abdominal pain (n=1). Patients with GI symptoms were significantly younger (median age: 60 years) as compared to patients without GI symptoms (median age: 68 years, p=0.01). There were a significantly higher number of patients with GI symptoms in the age group of 40-64 years than those without GI symptoms (56.8% vs. 32.5%, p=0.02). There were no significant differences in gender, race distribution, and smoking history between the two groups. There was no difference in baseline comorbidities between both groups, including hypertension, diabetes, chronic lung disease, chronic kidney disease, obstructive sleep apnea, atrial fibrillation, asthma, and liver cirrhosis. Majority of the patients had fever (65.4%) on admission. The distribution of abnormal vitals, including fever, heart rate ≥ 100 bpm, and respiratory rate ≥ 20 breaths per minute were also similar in both groups. Table 1 summarizes all the baseline characteristics of both groups with and without GI symptoms.

**Table 1:** Baseline demographics in patients with and without GI symptoms. HTN, hypertension; DM, diabetes mellitus; COPD, chronic obstructive pulmonary disease; CKD, chronic kidney disease; CAD, coronary artery disease; CVA, cerebrovascular accident; OSA, obstructive sleep apnea; ACE/ARB, angiotensin converting enzyme/angiotensin II receptor blocker; NSAID, nonsteroidal anti-inflammatory drug; SOB, shortness of breath

Characteristic	Patients without GI symptoms (n=40)	Patients with GI symptoms (n=44)	p-Value
Median age (IQR) – year	68.0 (22)	60.0 (22)	0.01
Age 18-39	1 (2.5)	4 (9.1)	0.2
Age 40-64	13 (32.5%)	25 (56.8%)	0.02
>=65	26 (65%)	15 (34.1%)	0.005
Male sex – no. (%)	27 (67.5%)	25 (56.8%)	0.31
Race – no. (%)			
Caucasian	26 (65.0%)	33 (75.0%)	0.57
African American	6 (15.0%)	4 (9.1%)	
Other	8 (20.0%)	7 (15.9%)	
BMI (kg/m^2^)	30.3 (10.7)	29.7 (8.9)	0.85
Current smoker – no. (%)	0 (0.0%)	3 (6.8%)	0.08
Former smoker – no. (%)	19 (47.5%)	16 (37.2%)	0.34
Comorbid conditions – no. (%)			
HTN	26 (65.0%)	30 (68.2%)	0.75
DM	14 (35.0%)	16 (36.4%)	0.89
COPD	6 (15.0%)	3 (6.8%)	0.22
CKD	5 (12.5%)	4 (9.1%)	0.73
CAD	8 (20.0%)	9 (20.5%)	0.95
Asthma	8 (20.0%)	10 (22.7%)	0.76
CVA	1 (2.5%)	0 (0)	0.47
OSA	6 (15.0%)	4 (9.1%)	0.5
Atrial fibrillation	2 (5.0%)	1 (2.3%)	0.5
Liver cirrhosis	5 (12.5%)	3 (6.8%)	0.37
ACE/ARB use	12 (30%)	21 (47.7%)	0.09
NSAID use	4 (10%)	10 (22.7%)	0.11
Symptoms on admission – no. (%)			
Fever	24 (60.0%)	31 (70.5%)	0.31
Fatigue	19 (47.5%)	23 (52.3%)	0.66
Sore throat	3 (7.5%)	4 (9.1%)	0.19
Cough	21 (52.5%)	32 (72.7%)	0.06
SOB	25 (62.5%)	32 (72.7%)	0.35
AMS	5 (12.5%)	5 (11.4%)	0.87
Vitals on admission			
Temperature, median (IQR) – ^0 ^F	99.0 (1.7)	99.0 (2.2)	0.74
Heart rate >100 beats per min – no. (%)	9 (22.5%)	12 (27.3%)	0.8
Respiratory rate > 20 breaths per minute – no. (%)	23 (57.5%)	24 (54.5%)	0.7

Patients With and Without Transaminitis (AST or ALT > 40 IU/L)

In patients with and without transaminitis, the baseline features, including age, race, and comorbidities, were similar in both groups. There was a significantly higher number of male patients with transaminitis than without transaminitis (79.5% vs. 42.5%, p=0.0001). All the baseline characteristics of patients with and without transaminitis are shown in Table 2.

**Table 2:** Baseline demographics in patients with and without transaminitis. HTN, hypertension; DM, diabetes mellitus; COPD, chronic obstructive pulmonary disease; CKI, chronic kidney disease; CAD, coronary artery disease; CVA, cerebrovascular accident; OSA, Obstructive sleep apnea; ACE/ARB, angiotensin converting enzyme/angiotensin II receptor blocker; NSAID, nonsteroidal anti-inflammatory drug

Characteristic	Patients without transaminitis (n=40)	Patients with transaminitis (n=44)	p-Value
Median age (IQR) – years	64.0 (21)	63.5 (26)	0.96
Male – no. (%)	17 (42.5%)	35 (79.5%)	0.001
Caucasian race – no. (%)	28 (70%)	31 (70.5%)	0.98
BMI (kg/m^2^)	30.5 (10.1)	29.7 (8.6)	0.72
African American – no. (%)	5 (12.5%)	5 (11.4%)	
Current smoker – no. (%)	1 (2.5%)	2 (4.5%)	0.61
Former smoker – no. (%)	19 (47.5%)	16 (37.2%)	0.34
Comorbidities – no. (%)			
HTN	26 (65.0 %)	30 (68.2%)	0.81
DM	17 (42.5%)	13 (29.5%)	0.25
COPD	7 (17.5%)	2 (4.5%)	0.06
CKI	5 (12.5%)	4 (9.1%)	0.73
CAD	9 (22.5%)	8 (18.2%)	0.62
Asthma	9 (22.5%)	9 (20.5%)	0.82
CVA	0 (0)	1 (2.3%)	0.33
OSA	5 (12.5%)	5 (11.4%)	0.87
Atrial fibrillation	1 (2.5%)	2 (4.5%)	0.61
Liver cirrhosis	2 (5.0%)	6 (13.6%)	0.17
ACE/ARB use	13 (32.5%)	20 (45.5%)	0.22
NSAID use	5 (12.5%)	9 (20.5%)	0.32
Vitals at presentation			
Temperature, median (IQR) – ^0 ^F	99.05 (2.9)	99.05 (1.3)	0.71
Heart rate >100 beats per min – no. (%)	8(20%)	13 (29.5%)	0.45
Respiratory rate > 20 breaths per min – no. (%)	22 (55%)	25 (56.8%)	0.86

Laboratory and imaging parameters

Patients With and Without GI Symptoms

The median white cell count was 5,800 and 6,700 per mm3 in patients with and without GI symptoms, respectively (p=0.17). The incidence of lymphopenia, thrombocytopenia, and elevated creatinine on admission was also similar in both groups. The median AST, ALT, and total bilirubin were also identical in both groups. The median CRP was 7.9 and 10.5 mg/dL in patients with and without GI symptoms, respectively (p=0.39). The distribution of ferritin, fibrinogen, interleukin-6, and D-dimer was not significantly different in both groups. There was also no significant difference in median peak AST, ALT, and total bilirubin between both groups. There was only one case each of concurrent influenza and respiratory syncytial virus in our study population. On imaging, majority (77.3%) of the patients had bilateral infiltrates, and imaging findings were also similar in these groups. Table 3 summarizes all the laboratory and imaging parameters of patients with and without GI symptoms.

**Table 3:** Laboratory and imaging findings in patients with and without GI symptoms. AST, aspartate aminotransferase; ALT, alanine aminotransferase; CRP, C-reactive protein; ULN, upper limit of the normal; ALP, alkaline phosphatase; PCR, polymerase chain reaction; CXR, chest X-ray; RSV PCR, respiratory syncytial virus polymerase chain reaction

Factor	Patients without GI symptoms (n=40)	Patients with GI symptoms (n=44)	p-Value
Laboratory Findings on Admission			
White cell count, median (IQR) – per mm^3^	6.7 (4.9)	5.8 (2.8)	0.17
Lymphocyte count, median (IQR) – per mm^3^	1.08 (0.77)	0.96 (0.91)	0.54
Lymphocyte <1,500, median (IQR) – per mm^3^	31 (77.5%)	33 (75%)	0.78
Platelet count, median (IQR) – per 10^9^/L	221.5 (115.5)	191.0 (101.3)	0.36
Serum creatinine, median (IQR) – mg/dL	1.03 (0.67)	0.96 (0.62)	0.59
Aspartate aminotransferase (AST), median (IQR) – U/L	37 (41)	38.5 (26)	0.7
Alanine aminotransferase (ALT), median (IQR) – U/L	28.5 (36)	23.0 (30)	0.43
Total bilirubin, median (IQR) - mg/dl	0.5 (0.4)	0.5 (0.4)	0.86
Troponin, median (IQR) - ng/dl	0.01 (0.02)	0.01 (0.01)	0.07
Troponin >0.03 ng/mL – positive/total no. (%)	9 (22.5%)	5 (11.4%)	0.17
IL-6, median (IQR) – pg/mL	19.0 (53.6)	29.0 (52.8)	0.65
CRP, median (IQR) – mg/dL	10.55 (12.6)	7.9 (9.5)	0.39
Ferritin, median (IQR) – ng/mL	652.2 (560.1)	602.1 (841.6)	0.68
Fibrinogen, median (IQR) – mg/dL	538 (287)	598 (283)	0.73
D-dimer, median (IQR) – ng/mL	1190 (2540)	985 (1921)	0.87
Peak labs during hospitalization			
Peak AST, median (IQR) – U/L	57.0 (73)	68.0 (87.5)	0.56
AST >1x ULN – no. (%)	25 (62.5%)	31 (70.5%)	0.49
Peak ALT, median (IQR) – U/L	54.0 (68)	68.0 (97)	0.49
ALT >1x ULN – no. (%)	24 (60.0%)	30 (68.2%)	0.43
Peak ALP, median (IQR) – U/L	90.5 (43)	83.0 (49)	0.76
ALP >123 IU/L – no. (%)	7 (17.5%)	10 (22.7%)	0.55
Peak Total Bilirubin, median (IQR) – U/L	0.55 (0.7)	0.7 (0.8)	0.65
Total bilirubin >1.3 mg/dL – no. (%)	8 (20.0%)	10 (22.7%)	0.76
Co-infection – no. (%)			
Influenza PCR	0 (0%)	1 (2.3%)	>0.99
RSV PCR	0 (0%)	1 (2.3%)	>0.99
CXR findings – no. (%)			0.82
Bilateral infiltrates	32 (80%)	33 (75%)	
Unilateral infiltrates	4 (10%)	5 (11.4%)	
No infiltrates	4 (10.0%)	6 (13.6%)	

Patients With and Without Transaminitis

In patients with transaminitis, there was no significant difference in total leukocytes count and lymphopenia as compared to patients without transaminitis. The median platelets count was significantly lower in patients with transaminitis (180 K/uL vs. 265 K/uL, p=0.04). Patients with transaminitis also had significantly elevated serum creatinine (1.01 mg/dL vs. 0.87 mg/dL, p=0.04) at the time of admission. Ferritin level on admission was almost doubled and significantly elevated in patients with transaminitis (median 746 ng/mL) as compared to patients without transaminitis (median 370 ng/mL, p=0.004). However, other laboratory parameters, including CRP, D-dimer, interleukin-6, and fibrinogen levels, were similar in both groups. The chest X-ray findings were also not different in both groups. These results are displayed in Table 4.

**Table 4:** Laboratory and imaging findings in patients with and without transaminitis. AST, aspartate aminotransferase; ALT, alanine aminotransferase; PCR, polymerase chain reaction; CXR, chest X-ray; RSV, respiratory syncytial virus;

Factor	Patients without transaminitis (n=40)	Patients with transaminitis (n=44)	p-Value
Laboratory values on admission			
White cell count, median (IQR) – per mm^3^	6.9 (4.2)	5.6 (2.4)	0.68
Lymphocyte count, median (IQR) – per mm^3^	1.00 (1.03)	0.78 (0.80)	0.53
Lymphocyte <1,500, median (IQR) – per mm^3^	29 (72.5%)	35 (79.5%)	0.44
Platelet count, median (IQR) – per 10^9^/L	265 (75.5)	180 (76)	0.04
Serum creatinine, median (IQR) – mg/dl	0.87 (0.35)	1.01 (0.64)	0.04
AST, median (IQR) – U/L	27.5 (12)	63 (41)	<0.001
ALT, median (IQR) – U/L	15.5 (7)	48.5 (34)	<0.001
Total bilirubin, median (IQR) - mg/dl	0.40 (0.5)	0.45 (0.3)	0.06
Troponin, median (IQR) - ng/dl	0.01 (0.01)	0.01 (0.02)	0.36
Interleukin-6, median (IQR) – pg/mL	48.9 (104.1)	29.5 (51.3)	0.55
C-reactive protein (mg/dL)	11.9 (12.08)	9.2 (10.4)	0.37
Ferritin, median (IQR) – ng/mL	370 (541)	746 (1161)	0.004
Fibrinogen, median (IQR) – mg/mL	627 (209)	548 (296)	0.28
D-dimer, median (IQR) – ng/mL	1850 (3325)	4834 (3940)	0.96
Peak labs during hospitalization			
AST, median (IQR) – U/L	40.5 (44.5)	79.0 (77.5)	0.004
ALT, median (IQR) – U/L	38.5 (62)	72.0 (99)	0.009
Total bilirubin, median (IQR) – mg/dL	0.55 (0.7)	0.7 (0.8)	0.13
Co-infection – no. (%)			
Influenza PCR	1 (2.5%)	0 (0)	0.29
RSV PCR	0 (0)	1 (2.3%)	0.33
CXR findings – no. (%)			
Bilateral infiltrates	33 (82.5%)	32 (72.7%)	0.54

Outcomes

Patients With and Without GI Symptoms

There were 59.5% (n=50) patients who required admission to the ICU during their hospitalization. ICU admissions were similar between both groups. Some 62% of the patients admitted to ICU required vasopressor support. Overall, vasopressor use and duration were not significantly different in both groups. The incidence of DVT was also similar in both groups. Almost half (48.8%) of our study population had CRS. Renal replacement therapy was required in 10.7% of patients, and rates were similar between both groups. Mechanical ventilation was required in 40.4% (n=34) of patients during their hospital admission. There was no difference in the need for mechanical ventilation between both groups (p=0.93). The duration of mechanical ventilation was also not different in both groups (7% vs. 9%, p=0.86). The mortality rate in our study population was 13% (n=11). Among them, six patients had no GI symptoms, and five patients had GI symptoms at presentation. There was no significant difference in mortality between both groups (p=0.62). These results are summarized in Table 5.

**Table 5:** Outcomes in patients with and without GI symptoms. GI, gastrointestinal; AKI, acute kidney injury; RRT, renal replacement therapy; CRRT, continuous renal replacement therapy; LOS, length of stay

Factor	Patients without GI symptoms (n=40)	Patients with GI symptoms (n=44)	p-Value
Admission to ICU – no. (%)	24 (60%)	26 (59.1%)	0.93
Circulatory shock requiring vasopressors – no. (%)	15 (37.5%)	16 (36.4%)	>0.99
Mean duration of pressor support, median (IQR) – day	4 (3)	5 (8)	0.35
Deep vein thrombosis – no. (%)	2 (5.0%)	5 (11.4%)	0.43
Cytokine release syndrome – no. (%)	16 (40.0%)	25 (56.8%)	0.13
AKI requiring RRT use (CRRT or dialysis) – no. (%)	3 (7.5%)	6 (13.6%)	0.48
Mechanical ventilation on admission – no. (%)	6 (15.0%)	7 (15.9%)	0.9
Mechanical ventilation anytime – no. (%)	16 (40%)	18 (40.9%)	0.93
Duration of mechanical ventilation, median (IQR) – day	11 (7)	9 (9)	0.82
LOS, median (IQR) – day	10.5 (13.7)	7.5 (8.25)	0.86
Mortality– no. (%)	6 (15.0%)	5 (11.4%)	0.62

Patients With and Without Transaminitis

There was no difference in the need for ICU admission, requirement, and duration of vasopressor support between both groups. There was a significantly higher number of DVT in patients with transaminitis than in patients without transaminitis (15.9% vs. 0, p=0.01). The incidence of CRS was also significantly higher in patients with transaminitis (59.1%) as compared to patients without transaminitis (37.5%) (p=0.04). The requirement of renal replacement therapy was similar in both groups. Patients with transaminitis were significantly likely to develop hypoxic respiratory failure (86.4% vs. 65.0%, p=0.02) and require mechanical ventilation (52.3% vs. 27.5%, p=0.02) as compared to patients without transaminitis. However, the duration of mechanical ventilation and length of stay in the hospital was not different between both these groups. There was a trend towards higher mortality in patients with transaminitis (18.2%) as compared to those without transaminitis (7.5%); however, it did not reach statistical significance (p=0.14) (Table 6).

**Table 6:** Outcomes in patients with and without transaminitis. DVT, deep vein thrombosis; CRS, cytokine release syndrome; AKI, acute kidney injury; RRT, renal replacement therapy; CRRT, continuous renal replacement therapy; AHRF, acute hypoxemic respiratory failure; LOS, length of stay

Factor	Patients without transaminitis (n=40)	Patients with transaminitis (n=44)	p-Value
Need for ICU admission -no. (%)	22 (55.0%)	28 (63.6%)	0.5
Circulatory shock requiring vasopressors - no. (%)	12 (30%)	19 (43.2%)	0.26
Mean duration of pressor support, median (IQR) – day	5 (11)	2 (6)	0.63
DVT - no. (%)	0 (0%)	7 (15.9%)	0.008
CRS - no. (%)	15 (37.5%)	26 (59.1%)	0.04
AKI requiring RRT use (CRRT or dialysis) - no. (%)	2 (5%)	7 (15.9%)	0.1
AHRF requiring oxygen - no. (%)	26(65%)	38 (86.3%)	0.022
AHRF requiring mechanical ventilation - no. (%)	11 (27.5%)	23 (52.3%)	0.02
Duration of mechanical ventilation, median (IQR) – day	13.5 (6)	10.0 (8)	0.18
LOS, median (IQR) – day	21 (15.2)	25.0 (13.25)	0.3
Mortality - no. (%)	3 (7.5%)	8 (18.2%)	0.14

Predictors of CRS, mechanical ventilation, and mortality

We performed multivariate logistic regression with an adjusted odds ratio (aOR) to identify predictors of CRS, mechanical ventilation, and mortality with a particular focus on GI symptoms and transaminitis on admission and during hospitalization. We adjusted for age, gender, various comorbidities including smoking history, hypertension, diabetes mellitus, chronic kidney disease, chronic obstructive pulmonary disease, asthma, coronary artery disease, obstructive sleep apnea, atrial fibrillation, and liver cirrhosis. After adjusting for these variables, presence of GI symptoms (aOR 4.2, 95% CI, 1.17-15.60, p=0.03), elevated AST on admission (aOR 4.7, 1.08-20.38, p=0.03), transaminitis on admission (aOR 5.69, 95% CI, 1.47-21.99, p=0.01), and elevated ALT during hospitalization (aOR 31.32, 95% CI, 3.75-260.94, p=0.001) were significantly associated with development of CRS.

The presence of either elevated AST or transaminitis on admission was also independently associated with the need for mechanical ventilation with aOR of 6.17 (95% CI, 1.49-25.44, p=0.02), and 9.26 (95% CI, 2.27-37.75, p=0.02) respectively. An elevated ALT during hospitalization was associated with an increased risk of mechanical ventilation with aOR of 6.74 (95% CI, 1.14-39.58, p=0.03). Also, total bilirubin > 1 x ULN was also independently associated with the need for mechanical ventilation with aOR of 7.29 (95% CI, 1.73-30.75, p=0.007).

**Figure 1:**
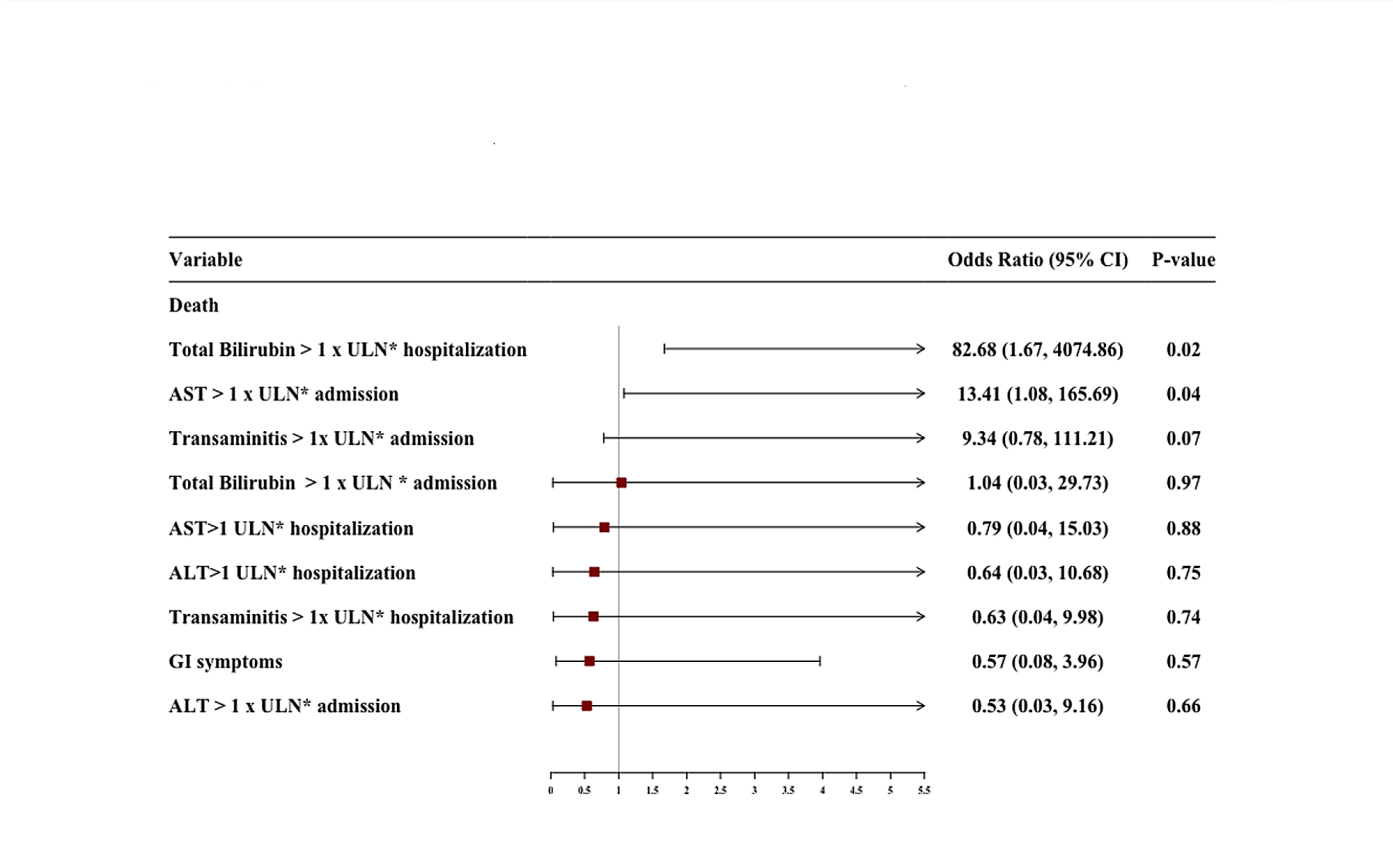
Forest plot of multivariate logistic regression showing predictors of mortality. .

**Figure 2:**
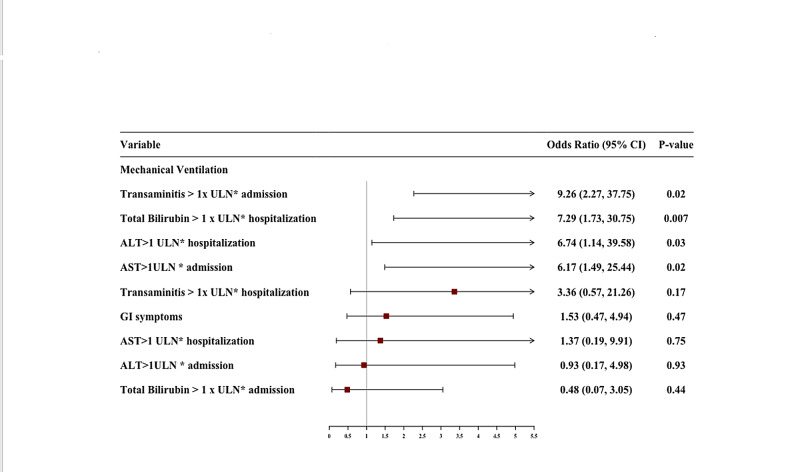
Forest plot of multivariate logistic regression showing predictors of mechanical ventilation.

**Figure 3:**
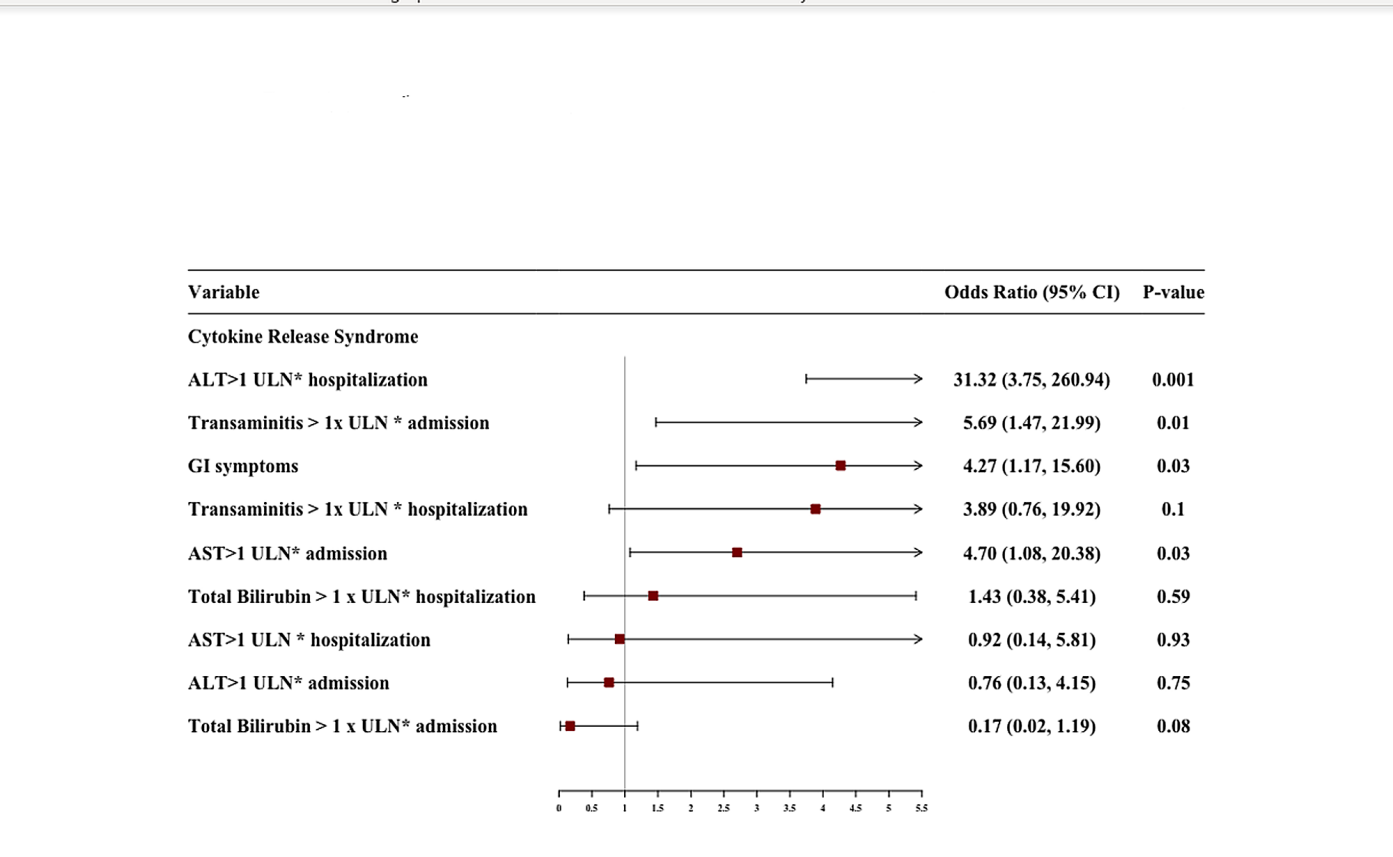
Forest plot of multivariate logistic regression showing predictors of CRS. CRS, cytokine release syndrome

An elevated AST on admission with aOR of 13.41 (95% CI, 1.08-165.69, p=0.04) and total bilirubin > 1 x ULN during hospitalization with aOR of 82.68 (95% CI, 1.67-4074.8, p=0.02) were independently associated with risk of death in COVID-19 patients. The results of multivariate analysis with aOR are shown in Table 7. The predictors of CRS, mechanical ventilation, and mortality after multivariate analysis are graphically displayed in Figures 1-3, respectively.

**Table 7:** Multivariate logistic regression showing predictors of mechanical ventilation, CRS, and mortality. CRS, cytokine release syndrome; GI, gastrointestinal; AST, aspartate aminotransferase; ALT, alanine aminotransferase; ULN, upper limit of the normal; OR, odds ratio

	Mechanical ventilation		CRS		Death	
Factor	Adjusted OR	p-Value	Adjusted OR	p-Value	Adjusted OR	p-Value
On admission						
GI symptoms	1.53 (0.47, 4.94)	0.47	4.27 (1.17, 15.60)	0.03	0.57 (0.08, 3.96)	0.57
AST > 1 x ULN	6.17 (1.49, 25.44)	0.02	4.70 (1.08, 20.38)	0.03	13.41 (1.08, 165.69)	0.04
ALT > 1 x ULN	0.93 (0.17, 4.98)	0.93	0.76 (0.13, 4.15)	0.75	0.53 (0.03, 9.16)	0.66
Transaminitis	9.26 (2.27, 37.75)	0.02	5.69 (1.47, 21.99)	0.01	9.34 (0.78, 111.21)	0.07
Total bilirubin > 1 x ULN	0.48 (0.07, 3.05)	0.44	0.17 (0.02, 1.19)	0.08	1.04 (0.03, 29.73)	0.97
During hospitalization						
AST > 1 x ULN	1.37 (0.19, 9.91)	0.75	0.92 (0.14, 5.81)	0.93	0.79 (0.04, 15.03)	0.88
ALT > 1 x ULN	6.74 (1.14, 39.58)	0.03	31.32 (3.75,260.94)	0.001	0.64 (0.03, 10.68)	0.75
Transaminitis	3.36 (0.57, 21.26)	0.17	3.89 (0.76, 19.92)	0.1	9.34 (0.78, 111.21)	0.07
Total bilirubin > 1 x ULN	7.29 (1.73, 30.75)	0.007	1.43 (0.38, 5.41)	0.59	82.68 (1.67, 4074.86)	0.02

## Discussion

To the best of our knowledge, this is the first study comparing the clinical course and outcomes of COVID-19 patients with and without gastrointestinal and hepatic manifestations in the U.S. population. Our study revealed that 52% of our patients who tested positive for SARS-CoV2 had at least one GI symptom at presentation. Our findings are in line with the studies by Pan et al. [9] and Zhang et al. [2], who reported an incidence of 50.7% and 39.6% for GI symptoms in their COVID-19 patients, respectively. Diarrhea was the most common GI symptom followed by nausea/vomiting and loss of appetite. Only one patient had abdominal pain in our study population. As such, our findings add to the evolving evidence that SARS-CoV2 might cause acute gastritis and enteritis.

Interestingly, a higher proportion of younger patients aged 40-64 years had GI symptoms when compared to more respiratory symptoms in patients aged > 65 years. This corroborates with the observation by Lin et al. [10], where the mean age of patients with GI symptoms was 48 years. Moreover, a higher proportion of our patients also had elevated AST or ALT at presentation. On multivariate analysis, the presence of transaminitis on admission was associated with a higher likelihood of developing ARDS requiring mechanical ventilation and CRS. Notably, an elevated AST on admission and elevated total bilirubin during hospitalization were associated with significantly higher mortality.

Previously published studies from China reported a higher likelihood of severe or critical illness in patients with GI symptoms. In a study by Jin et al. [5], a significantly higher percentage of patients with GI symptoms required admission to the ICU, were on mechanical ventilation, and developed transaminitis during hospitalization. A recent metanalysis of 35 studies by Mao et al. [8] suggested that COVID-19 patients with GI symptoms had a higher risk of developing severe disease. Despite the high incidence of GI symptoms in our patients, their presence did not seem to have any significant impact on the overall outcomes. There was no difference in the need for mechanical ventilation or mortality among patients with and without GI symptoms, except for an increased incidence of CRS. These findings are relatively similar to a large study from New York, who suggested that the presence of GI symptoms was associated with less severe disease and had no significant impact on the disease outcomes on multivariate analysis [7]. As such, it appears that the presence of GI symptoms in the American population might not have a strong association with a worsening disease when compared to the Asian population.

In addition to the GI tract involvement, hepatic manifestations have also been commonly reported in patients infected with SARS-CoV2. Recent studies have suggested a varying degree of liver enzymes elevation in patients with COVID-19, with a reported incidence ranging from 14.8% to 78% [11-14]. In a study from China, 78% of the patients with severe disease had increased transaminase levels [15]. They further suggested a strong association with increased AST or ALT with higher mortality. The authors suggested that there seemed to be a higher prevalence of liver injury in patients with severe disease when compared to those with mild disease. Interestingly, Jin et al. [5] also found a higher incidence of transaminitis in patients with GI symptoms, which could have indirectly caused worsening disease activity. Thus, data from all these studies does highlight the fact that the occurrence of abnormal liver function tests during hospitalization for COVID-19 is quite common and clinically significant liver injury or liver failure is rare [16-18]. However, it is still unclear if the elevated transaminases during hospitalization are indeed a surrogate clinical maker for higher levels of viremia.

An article describing the risk factors associated with hepatic injury in COVID-19 patients suggested the presence of lymphopenia and high CRP level to be independent risk factors for liver enzymes abnormalities [19]. Although the underlying mechanism of liver injury remains unclear, various direct and indirect etiologies have been postulated thus far. These include the cytopathic effect of the virus [20], hypoperfusion following circulatory shock [21], presence of multiorgan failure [22], drug-induced liver injury [23], and T-cell activated inflammatory responses such as cytokine release storm from SARS-CoV2 [24]. Other less unlikely but possible etiologies are hepatic congestion secondary to the use of positive pressure ventilation [20], ischemia, and infectious myositis. As such, the presence of elevated liver enzymes during hospitalization could be multifactorial and cannot be attributed to the primary spectrum of coronavirus disease. In our study, we attempted to primarily evaluate the implications of abnormal liver enzymes that occurred at the time of diagnosis, than during treatment or hospitalization. 

We evaluated for the presence of abnormal liver enzymes at presentation, thereby excluding the effect of external factors such as antiviral medications (lopinavir/ritonavir) [21], IL-6 inhibitors (tocilizumab) [25], antipyretics (acetaminophen) [6], antibiotics (macrolides, quinolones), steroids, positive pressure ventilation, etc. Additionally, the baseline comorbidities, including cirrhosis, first recorded laboratory parameters, and oxygen requirements on admission were not significantly different among our patients, thus eliminating the significant heterogeneity among our study groups. With the current emphasis on streamlining triaging efforts and healthcare resource utilization, we tried to perform a more detailed analysis of the clinical manifestations, hospital course, and outcomes of COVID-19 patients with and without GI and hepatic manifestations. Furthermore, we performed a robust statistical analysis by adjusting for comorbidities to decrease potential confounders.

Our study has several limitations, apart from having a single-center retrospective observational design. First, our findings cannot be widely generalized due to the relatively small study population and the rapidly changing demographic and clinical pattern of SARS-CoV2. Second, our study population represents our very first encounter with COVID-19 patients who were treated with medications that are currently under investigation with no clear, proven efficacy. Third, due to the short duration of our study period, we could not assess the factors associated with prolonged length of stay and readmissions in these patients. Lastly, although majority of our patients had hypertransaminasemia at presentation, we could not elucidate the primary cause of this phenomenon to guide the best treatment.

## Conclusions

In conclusion, the characteristics, clinical course, and outcomes of COVID-19 patients with GI symptoms were not worse when compared to those without any GI symptoms in our western study population. Importantly, we found a significantly high risk of developing ARDS requiring mechanical ventilation and CRS in COVID-19 patients with transaminitis at presentation. Notably, patients with an elevated AST on admission had a higher risk of mortality. Elevated total bilirubin anytime during hospitalization not only had a significantly higher likelihood of requiring mechanical ventilation but was also associated with higher mortality. Thus, frontline clinicians and first responders might consider evaluating for transaminitis in their preliminary laboratory assessments for making decisions regarding initial triaging and closer follow up. Prospective studies with a larger number of patients are required to further validate our observations.
